# The relationship between self-harm and bullying behaviour: results from a population based study of adolescents

**DOI:** 10.1186/s12889-021-10555-9

**Published:** 2021-03-31

**Authors:** Ingri Myklestad, Melanie Straiton

**Affiliations:** Department of Mental Health and Suicide, Norwegian Institute of Public Health, Skøyen, PO Box 222, 0213 Oslo, Norway

**Keywords:** Self-harm, Bullying behaviour, Depression, Parental support, Adolescents

## Abstract

**Background:**

This study aims to better understand the association between bullying behaviour (the bullied, the bullies and the bully-victims) and self-harm, and which protective factors moderate this association.

**Methods:**

A total of 16,182 adolescents, aged 12 to 19 years, were invited to participate in the cross-sectional Ung-data survey. This survey covered various aspects of young people’s lives. The response rate was 87%. To assess the relationship between self-harm and bulling behaviour, and psychological- and environmental covariates, we conducted logistic regression analyses. In addition, we tested for potential interaction effects between protective factors and the three bullying groups on self-harm.

**Results:**

Fifteen percent of participating adolescents reported engaging in self-harm during the last year. The risk of self-harm was six times higher for the “bully-victims”, five times higher for the bullied, and three times higher for the bullies, compared to the “neither-bullied nor bullies”. The risk of self-harm in the face of being bullied was significantly greater for girls than boys. Depression, anxiety and parental conflict accounted for some of the association between being bullied and self-harm, and between bully-victims and self-harm. School behavioural problems accounted for some of the association between the bullies and self-harm and the bully-victims and self-harm. The relationship between the bullied and self-harm was significantly moderated by parental support and school well-being, while the relationship between “bully-victims” and self-harm was moderated by school well-being.

**Conclusion:**

There is a strong link between bullying and self-harm. Interventions to address bullying may reduce self-harm. Our findings also suggest that high levels of parental support and school well-being may buffer the harmful relationship between bullying behaviour and self-harm. Addressing these factors may be important in reducing the risk of self-harm among those experiencing bullying.

## Background

Self-harm is a major public health problem [1] and adolescents are particularly at risk [2]. In a review of 52 studies on self-harm, the international lifetime estimate of self-harm among adolescents was 17% [3], and the 12-month prevalence was 10–19% [4]. A recent Norwegian study from 2020 showed that the 12-month prevalence among young adolescents (13–15 years) was 16.2% and that the prevalence had increased 4-fold over a 15-year period [5]. Girls engage in self-harm more than boys [3, 5, 6]. A study from seven different countries showed that both the lifetime and the 12- month prevalence of self-harm was three times larger among adolescent girls compared to adolescent boys [7].

Previous research suggests that bullying victimisation is a risk factor for self-harm [8–10]. Yet, not all adolescents who experience bullying harm themselves. This study aims to better understand the link between bullying and self-harm.

Consensus on the definition of self-harm is lacking in the literature. Common definitions include deliberate self-harm (DSH), that refers to intentional self-poisoning or self-injury, and non-suicidal self-injury (NSSI) that refers to the deliberate and direct injury of one’s own body tissue without suicidal intent, such as scratching, cutting, hitting and burning oneself [11, 12]. However, the suicidal intention of self-harm is often unknown, and we do not know how individuals, particularly adolescents, understand the term self-harm. In this study, we therefore use the NICE Clinical Guidelines [13] definition of self-harm: ‘self-poisoning or self-injury, irrespective of the apparent purpose of the act’, that is a shorter and broader definition of the World Health Organization WHO/EURO multicentre study [14].

Risk factors for self-harm include a complex interplay of genetic-, biological-, mental disorder, psychological- and environmental factors [1]. Psychological factors include depression, anxiety, emotional dysregulation, personality disorders, aggression, and low self-esteem [15]. Environmental factors include exposure to life stress events such as sexual abuse, violence, early parental separation loss, bullying, family/parental conflicts, and school academic problems [16, 17]. Fewer studies have however explored protective factors for self-harm, though social support from community, family and friends, and personal factors (social competency, problem-solving skills and positive temperament) may protect against self-harm [18]. Studies among adolescents indicate that social support and connectedness to family, peers, and school, and personal factors such as high self-control are associated with reduced risk of self-harm [19, 20].

Many studies confirm that bullying victimisation, which refers to being a victim of aggressive behaviour or intentional harm, repeatedly over time, and which involves an imbalance in power [21], is associated with self-harm [6, 9]. Verbal bullying by peers, in particular, seems to have a strong association with self-harm among adolescents [22]. O’Conner [6] found that girls who were bullied were three times as likely to engage in self-harm as others, while bullied boys were twice as likely. Cyberbullying, that is bullying through electronic forms of contact such as mobile phones and the internet, have emerged in recent years [23]. A review article [24] examining the relationship between bullying victimisation and self-harm showed a significant association between self-harm and both victims of cyber-bullying and traditional bulling face- to face. The co-occurrence of traditional- and cyber-bullying victimisation also showed a strong association with self-harm.

One explanation for the strong association between bullying victimisation and self-harm may be that both are associated with emotional problems such as anxiety and depression [25]. Bully victimisation is linked to increased feelings of anxiety and depression, and several studies have shown that self-harm can, among other functions, work as an affect-regulation function by decreasing negative emotions [26–28]. Thus, negative emotions may mediate the relationship between bullying victimisation and self-harm [28, 29]. In addition, a large study of Irish adolescent boys showed that school-related problems, physical abuse, worries about sexual orientation and serious conflicts with parents heighten the risk of self-harm among those who were bullied [10].

Few studies, however, appear to have investigated possible protective factors for self-harm in the face of bullying. Studies that have, found that family- and parental support and an authoritative parental style [9] moderate the relationship between self-harm and bullying victimisation [10, 20] and one study found that parental support also moderated the relationship between self-harm and bully aggressors [20]. There appears to be a lack of research investigating protective factors outside of the family such as support from friends, and school well-being (including teacher support). These protective variables could potentially have a moderating effect on the relationship between self-harm and the different types of bullying behaviours.

Interestingly, studies indicate that it is not only the experience of being the victim of bullying that is associated with self-harm, but also the experience of being a bully aggressor [20]. Adolescents who bully are at increased risk of aggressive and delinquent behaviours, school failures, and dropping out of school on the one hand, but also at increased risk of depression and suicidal ideation on the other [30, 31]. A recent literature review [24] presented a meta-analysis of 14 studies to examine the association between bully aggressors and self-harm in adolescents. The results showed the odds of self-harm were almost doubled when youth reported bullying perpetration. Furthermore, Barker and colleagues also found that bully aggressors had an increased risk of engaging in self-harm, especially if they also had a history of being bullied, so-called “bully-victims” [30]. However, little is known about what risk- and protective factors may confound the relationship between the different types of bulling and self-harm.

In this study, we investigated the relationship between self-harm and 1) being bullied (“bullied”), 2) bullying others (“bullies”) and 3) both being bullied and bullying others (“bully-victims”), and which factors might account for these relationships. Furthermore, we aimed to identify which protective factors may moderate the relationship between these bullying behaviours and self-harm.

### Hypotheses

#### We hypothesised that

- The three bullying behaviours: 1) being bullied (“bullied”), 2) bullying others (“bullies”) and 3) both being bullied and bullying others (“bully-victims”) would be associated with higher odds of self-harm compared to those who were neither bullied nor bullies.
- Gender (being a girl), socioeconomic differences (having a poor socioeconomic background), having school behavioural problems, experiencing parental conflict and having emotional problems (depression and anxiety) would be variables negatively associated with self-harm (potential risk factors). Controlling for these variables would account for some of the association between self-harm and the three bullying groups.
- Social support from parents, parental monitoring, social support from friends, friendship and school well-being (including teacher support) would protect against self-harm. Controlling for these factors would account for some of the association between self-harm and the three bullying groups.
- The protective factors would moderate the relationships between the three bulling groups and self-harm (interaction effects).

## Methods

### Study design and participants

“Ungdata” is a cross-sectional, large, national survey, designed for adolescents and conducted at the municipal level in Norway [32]. “Ungdata” covers various aspects of young people’s lives, such as relationships with parents and friends, leisure activities, health issues, local environment, well-being, and school issues. The survey also includes questions about tobacco and drug use, and participation in violence, bullying and self-harm. We were granted access to the “Ungdata” from the Norwegian Social Research Institute (NOVA). NOVA is responsible for the national coordination of the project, while the regional Drug and Alcohol Competence Centres are responsible for conducting the municipal surveys.

Participants in the “Ungdata” study were high-school pupils and students (grades 8th–13th, 50% girls) from 85 different municipalities in Norway, conducted in “the Ungdata” survey in 2014 (*N* = 47,450) [32, 33]. The response rate was 84% among the younger high school pupils (grades 8th–10th) and 66% among the older high school students (grades 11th–13th) [33]. All participants filled in an online questionnaire during school hours. The questionnaire consisted of both a core part, which is identical for all municipalities, and an elective part, with questions that municipalities could choose, based on interest and need. Questions on self-harm were in the elective part of the questionnaire. Thus, only adolescents living in the 23 municipalities that had chosen to include this part of the questionnaire could respond (*N* = 16,182). A total of 14,093 adolescents answered the questions relating to self-harm, yielding a response rate of 87%.

A public health coordinator in each municipality administered the survey, and local contacts in each school approached the adolescents together with teachers. The local contacts and teachers ensured that survey procedures were followed and that adolescents did not collaborate while responding to the survey. All the municipal surveys were conducted anonymously. Participation was voluntarily. Parents were informed about the surveys and given the opportunity to withdraw their children from participation [34]. The data were cleaned by NOVA Ungdata according to their criteria to remove non-serious responses prior to issuing the data. Details of the criteria can be found in “the Ungdata” report [32]. Because the dataset was conducted without personal identifiable information, specific ethical approval was not required for this study and was waved by the Norwegian Centre for Research data (NSD). We conducted the analyses in accordance with the NSD’s data protection regulations.

### Measures

#### Self-harm

Self-harm was the dependent variable. To measure self-harm, participants were asked: (1) “Have you ever tried to harm yourself?” Those who responded yes, were then asked: (2) “Have you tried to harm yourself in the past 12 months?”. We wanted specifically to study those who had been self-harming the last year. This was because those who have self-harmed in the last 12 months are most at risk in the future, and the timeline was more aligned with the bullying measure (see below for the bullying measure). Thus, we grouped adolescents into two categories: Those who had self-harmed in the past 12 months (value 1) and those who had not self-harmed in the last 12 months (regardless of if they had harmed themselves prior to this) (value 0).

#### Bullying behaviour

Bullying behaviour was a categorical independent variable. Bullied by peers was measured by combining two questions: “Are you sometimes teased, threaten, or frozen out by other young people in school or in your free time?” and “Are you sometimes teased, or threaten, by other young people online or on your mobile phone?” Response options for both questions were: (1) Yes, several times a week, (2) Yes, around once a week, (3) Yes, around once a fortnight, (4) Yes, once a month, (5) Almost never (6) Never. Those who responded with 1 to 4, on both, or one of the questions, were categorised as: “Bullied by peers” and valued 1, and those who responded with 5 or 6 on both questions were categorised as “Not bullied by peers” and valued 0. Bullying other peers was measured by combining the following two questions: “Do you sometimes take part in teasing, threatening or freezing out other young people at school or in your free time?” and “Do you sometimes take part in teasing, and/or threatening other young people online or by mobile phone?” Response options for both questions were: (1) Yes, several times a week, (2) Yes, around once a week, (3) Yes, around once a fortnight, (4) Yes, once a month, (5) Almost never (6) Never. Adolescents who responded with 1 to 4, on both, or one of the questions, were categorised as “Bullying other peers” and valued 1, and adolescents who responded with 5 or 6 on both questions were categorised as “Have not bullied other peers” and valued 0. We then created a new variable combining the variables “Bullied by peers” and “Bullying other peers”. First, those who were neither “Bullied by peers” nor “Bullying other peers” were coded with 0. Those who had been “Bullying other peers” but were not “Bullied by peers” were coded with 1 (“bullies”). Those who were both “Bullied by peers” and “Bullying other peers” were coded with 2 (“bully-victims”). Finally, those who were “Bullied by peers”, but not “Bullying other peers” were coded with 3 (“bullied”).

#### Gender

Gender was a dichotomous variable with Boys (0) and Girls (1).

#### Social support of parents and social support of friends

The measure “Willingness to seek social support” was based on Sarason’s social support measure [35]. We used two separate questions, one referring to parents and one to friends. Participants were asked: “Imagine that you have a personal problem. You feel sad and need someone to talk to. Who would you talk to, or ask for help? A) Parents? B) Friends? Response options were: (1) Definitively, (2) Maybe and (3) No. For each support person, we combined response options (1) “Definitively” and (2) “Maybe”, with “Yes” (coded with two), while No was coded with 1.

#### Friendship

Friendship was measured with the question: Do you have at least one friend who you trust completely and can tell absolutely anything to? Response options: 1) Yes, I am sure, 2) Yes, I think so, 3) I do not think so, and 4) I have no one I could call a friend now a days”.

#### Father- and mother education

Father/Mother education was measured by responses to the questions: a) “Does your father have university or college level education?” (1) Yes (2) No and b) “Does your mother have university or college level education?” (1) Yes (2) No.

#### Psychological distress: symptoms of depression and anxiety

The measures of symptoms of depression and anxiety were short versions of the scales: “Hopkins Symptom Checklist” (HSCL) [36, 37] and “Depressive Mood Inventory” [38]. Earlier studies have shown that short version of HSCL has good validity [39, 40]. Participants were asked: «During the past week, have you been affected by any of the following issues: 1)” Felt that everything is a struggle”; 2)” Had sleep problems”; 3)” Felt unhappy, sad or depressed”; 4)” Felt hopelessness about the future”; 5)” Felt stiff or tense”; 6)” Worried too much about things”; 7)” Suddenly felt scared for no reason”; 8)” Felt constant fear or anxiety”; 9)” Felt exhausted or dizzy”; 10)” Been nervous or felt uneasy”; 11)” Been easily moved to tears” and 12)” Tended to blame yourself for things”. Each question had four response categories: (1) Not at all, (2) Not much; (3) Quite a lot; and (4) A great deal. We then conducted a Principal components factors analysis (PCA) with oblique rotation. Based on eigenvalues and scree-plots, we found two factors: questions 1–6 (Depression) and questions 7–12 (Anxiety). A mean score index was then calculated for depression and anxiety. Cronbach’s alpha was 0.9 for each of the factors, indicating a satisfactory reliability of each measure.

#### Family’s financial situation

Family’s financial situation was based on responses to the question: “Financially, has your family been well off or badly off, over the past 2 years? The response categories were: (1) “We have been well off the whole time”, (2) “We have generally been well off”, (3) “We have neither been well off or badly off” (4) “We have generally been badly off” and (5) “We have been badly off the whole time”.

#### School behavioural problems

School behavioural problems (SBP) was measured by four items from the conceptual domain of the “School-related problem behaviour” in the “Bergen questionnaire on antisocial behaviour” [41, 42]. This measure has been used in earlier Ungdata-studies (Young in Norway studies) in 1992, 2002, and 2010, and the scale was shortened down when the questionnaire was revised in 2013 [32]. The measure included: “Have you done or experienced any of the following things during the past 12 months?”:” Had a big argument with a teacher”;” The school have contacted your parents for something bad you did”,” Skipped school” and “Been in a fight (without weapon)”. The response options were: (1) “Never”; (2) “Once” (3) “2–5 times”; (4) “6–10 times” and 5)“11 times or more”. PCA with oblique rotation was conducted for the four questions. Based on eigenvalues and scree-plots, the items loaded on one factor. A mean score was calculated for the four items. Cronbach’s alpha was 0.7, indicating a satisfactory level of reliability for school behavioural problems.

#### Relationship with parents: parental monitoring and parental conflict

This measure is based on elements of the concept of parenting style [43], particularly authoritative parenting [44, 45], from the “strictness-supervision scale”, which assesses parental monitoring and limit setting. In addition, some of the items measured the concept “conflicts with or between parents” [32]. The adolescents responded to the following questions: “Below are some statements that may describe your relationship with your parents”: a) “My parents usually know where I am, and who I am with, in my free time”; b) “My parents know most of the friends I hang out with in my free time”, c) “I try to hide what I do in my free time from my parents”; d)“My parents know my friends’ parents”; e) “I often argue with my parents”, f)“The adults in my family often argue” and g) “My parents know who I am in touch with on the internet”. Response were given on a 4-point scale from: (1) Very true; (2) Quite true; (3) Not very true, and (4) Not at all true. We conducted a PCA with oblique rotation on the seven statements. Based on eigenvalues and scree plots, two factors were extracted: “Parental monitoring” (items a, b, d and g) and “Parental conflict” (items c, e and f). A mean score index was calculated for each and Cronbach’s alpha showed a satisfactory level of reliability (0.7) for both concepts; (“Parental monitoring”, α = 0.7 and “Parental conflict”, α = 0.7) [46].

#### School well-being

The questions measuring school well-being have previously been used in studies of young people in Norway [32], and include the following: “Do you agree or disagree with the following statements about your situation at school:, “I enjoy school”, “I feel that I fit in with the students at my school”, “I often do not want to go to school”, “My teachers care about me”, and “I am bored at school”. Evaluation of the statements was on a 4-point scale from: (1) “Totally agree”, (2) “Somewhat agree”, (3) “Somewhat disagree” to (4) “Totally disagree”. To measure how the items correlated with each other and if they fitted together, a PCA with oblique rotation of the five statements was conducted. Based on eigen-values and screeplots, only one factor was extracted. A mean index score was calculated for the five items after reversing responses in the first, second and forth items so a high score represented good school well-being. Cronbach’s alpha was 0.72, indicating a satisfactory level of reliability.

### Statistical analyses

To test significant differences between those who had self-harmed during the last 12 months and those who had not self-harmed during the last 12 months, we used chi-square tests for all categorical variables. Independent-samples t-tests were conducted to compare the continuous variables. To assess the relationship between the dichotomous dependent variable/the outcome variable: “self-harmed last 12 months”, (yes/no), and the independent variable (bulling behaviour) and the covariates (gender, depression, anxiety, family’s financial situation, mother/father education, school problems, parental conflict, parental monitoring, school well-being, parental support, friend support and friendship), we conducted logistic regression analyses. Gender, bulling behaviour, parental support, friend support and friendship were treated as categorical variables. We conducted bivariate logistic regression analyses for the dichotomous dependent variable “self-harmed last 12 months”, and the independent variable (bullying behaviour) and each covariate separately. Then we ran various logistic regression models, with the addition of the covariates in order to evaluate which factors would reduce the strength of the association between bullying behaviour and self-harm. We did this first for all potential risk factors and then for all potential protective factors. To determine whether the potential protective factors moderated the relationship between bullying behaviour and self-harm, we performed separate regression analyses, adding one interaction term at a time. All continuous variables in the interaction terms were first standardised [47]. We then plotted the values of the unstandardised regression coefficients (including intercept) and means and standard deviations of the independent variables, moderators and the interaction terms in the cells indicated in the Jeremy Dawson’s excel sheet, http://www.jeremydawson.com/slopes.htm [47–49]. This was to aid interpretation and to visualise the significant interaction effects.

## Results

### Descriptive statistic

Of the 14,093 adolescents who responded to the questions on self-harm, 15.3% (*n* = 2149) had engaged in self-harm during the last 12 months, with more girls (22.5%) than boys (8.0%). Around 11% of the sample reported that they had experienced being bullied by their peers (the “bullied”), at least once a month. Three percent had been both victims of bullying and bullied others (the “bully-victims”) and 2% had bullied others but not been bullied themselves (the “bullies”).

Table 1 shows significant differences in self-harm for the categorical variables in the study (gender, bullying behaviour, friendship, social support and parental education). We also found significant differences between those who had self-harmed and those who had not in scores for all continuous variables (depression, anxiety, family’s financial situation, school behavioural problems (SBP), parental conflict, parental monitoring, and school well-being).

**Table 1:** Significant differences between demographic-, psychological- and environmental variables among adolescents who self-harmed and not self-harmed (*N* = 14,093)

Variables	Frequencies *n*	Self-harm *%*		Chi-square *χ*^*2*^	*df*
		Yes	No		
Self-harm	14,093	15.3	84.7		
Categorical variables					
Gender					
Female	7026	22.5	77.5	569.7***	1
Male	6970	8.0	92.0		
Bullying behaviour					
Neither bullied nor bullies	11,528	10.9	89.1		
Bullied	1526	38.1	61.9		
Bullies	318	28.0	72.0		
Bully-victims	401	42.1	57.9	1054.0***	3
Friendship					
Yes	12,465	14.0	86.0		
No	1437	26.0	74.0	142.9***	1
Social support friends					
Yes	11,878	14.5	85.5		
No	1699	21.2	78.8	52.0***	1
Social support parents					
Yes	11,027	10.3	89.7		
No	2527	36.3	63.7	1086.6***	1
Education, Mother					
High	8990	25.0	75.0		
Low	3220	34.3	65,7	67.9***	1
Education, Father					
High	7896	32.4	67.6		
Low	3988	40.1	59.9	38.9***	1
Continuous variables		*M* (*SD*)		*t-test*	*Range*
Depression		21.7 (6.3)	13.4 (4.8)	−56.9***	8–32
Anxiety		13.6 (5.2)	7.9 (2.8)	−49.4***	6–24
SBP		5.1 (2.7)	3.7 (1.4)	−23.0***	3–15
Parental conflict		6.5 (2.3)	4.9 (1.7)	−30.3***	3–12
Family’s financial situation		2.4 (1.1)	1.9 (0.9)	−20.0***	1–5
School well-being		13.5 (3.2)	16.4 (2.4)	38.7***	5–20
Parental monitoring		11.0 (2.8)	12.6 (2.3)	24.1***	4–16

Note. Significant result = *p < 0.05, **p < 0.01, and ***p < 0.001. *M* Mean. *SD* Standard Deviation. Self-harm = Self harmed last 12 months. Bullying behaviour = Being bullied by peers at least one time a month last 12 months (the bullied)or bullying other peers at least one time a month last 12 months (bullies), or both bullying others and being bullied by peers at least one time a month last 12 months (bully-victims). SBP=School behavioural problems. Chi-square test was used to test significant differences for categorical variables, and independent sample t-test was used to test significant differences for the continuous variables

### Risk factors

Table 2, Model 1 shows that the odds of self-harm was highest among the “bully-victims”. The odds ratio (OR) was six times higher for this group compared with those who were neither bullied nor bullies themselves. Additionally, the odds of self-harm were 5.0 times higher among “bullied” adolescents and 3.2 times higher among the “bullies”, compared with those who were neither bullied nor bullies themselves.

**Table 2:** OR of self-harm last year on bullying behaviour, demographic-, psychological- and environmental independent variables

	Model 1	Model 2	Model 3	Model 4	Model 5	Model 6
	OR (95% CI)	OR (95% CI)	OR (95% CI)	OR (95% CI)	OR (95% CI)	OR (95% CI)
Neither bullied nor bullies	1.0 (ref.)					
Bullied	5.04 (4.47–5.68)***	4.94 (4.36–5.59)***	4.26 (3.69–5.0)***	2.30 (1.95–2.71)***	2.18 (1.85–2.58)***	1.97 (1.66–2.34)***
Bullies	3.19 (2.48–4.09)***	4.43 (3.40–5.77)***	3.52 (2.56–4.84)***	2.12 (1.47–3.05)**	2.16 (1.48–3.14)**	1.49 (1.01–2.22)*
Bully-victims	5.97 (4.86–7.35)***	8.28 (6.65–10.34)***	7.32 (5.60–9.56)***	3.24 (2.39–4.38)***	2.80 (2.04–3.85)***	1.82 (1.29–2.55)**
Gender	3.34 (3.02–3.71)***	3.73 (3.34–4.17)***	3.55 (3.12–4.04)***	2.16 (1.88–2.49)***	1.72 (1.49–2.00)***	1.94 (1.66–2.34)***
Family’s financial sit.	1.60 (1.53–1.68)***		1.39 (1.31–1.47)***	1.12 (1.05–1.19)**	1.12 (1.04–1.19)**	1.04 (0.97–1.12)
Education mother	1.56 (1.41–1.74)***		1.25 (1.08–1.44)**	1.21 (1.03–1.41)**	1.22 (1.04–1.43)**	1.18 (1.00–1.38)
Education father	1.39 (1.26–1.55)***		1.04 (0.91–1.19)	0.99 (0.85–1.15)	0.99 (0.85–1.15)	0.97 (0.83–1.13)
Depression	1.23 (1.22–1.24)***			1.20 (1.18–1.21)***	1.12 (1.10–1.13)***	1.11 (1.08–1.11)***
Anxiety	1.33 (1.31–1.34)***				1.14 (1.12–1.17)***	1.12 (1.12–1.16)***
Parental conflict	1.44 (1.41–1.48)***					1.15 (1.11–1.19)***
School problem	1.37 (1.34–1.40)***					1.15 (1.11–1.19)***

Note. *p < 0.05 **p < 0.01 ***p < 0.001, *OR* Odds ratio, Family’s financial sit. = Family’s financial situation. Model 1 shows crude/unadjusted OR, bivariate associations with no controls, Model 2 shows adjusted OR with control for bullying behaviour and gender, Model 3 shows adjusted OR with control for bullying behaviour, gender and socioeconomic background. Model 4 shows adjusted OR with control for bullying behaviour, gender, socioeconomic background and depression. Model 5 shows adjusted OR with control for bullying behaviour, gender, socioeconomic background, depression, and anxiety, Model 6 shows adjusted OR with control for all independent variables included in the table (bullying behaviour, gender, socioeconomic background, depression, anxiety, parental conflict and school problem)

The bivariate analysis (Table 2, Model 1), showed that low socioeconomic background (poor family financial situation and low parental education), depression, anxiety, parental conflict, and school behavioural problems (SBP) were significantly related to self-harm. Those whose parents did not have higher education, who had a poorer family financial situation and higher scores on depression, anxiety, parental conflict, and school behavioural problems (SBP) had higher odds of self-harm. The odds of self-harm among girls was 3.34 times higher than for boys. The OR for gender remained stable and increased a little to 3.73 when controlling for bullying behaviour (see Table 2, model 2).

In Model 3, we controlled for socioeconomic background, gender and bullying behaviour. There were small reductions in OR for the three groups of bullying behaviour from model 2 to model 3. Thus, socioeconomic status accounted for little of the relationship between bullying behaviour and self-harm.

Adding depression to the model reduced the odds of self-harm more than 10% from model 3 to model 4 for all three types of bullying behaviours. Thus, depression accounted for part of the relationship between bullying behaviours and self-harm for all three types of bulling behaviour [50]. Adding anxiety to model 5, the odds ratio of self-harm reduces less than 10% from model 4 to model 5 for the bullies and thus anxiety do not account for the relationship between the bullies and self-harm.

In the final model (Model 6), we controlled for all predictors. The OR for self-harm was significant for depression, anxiety, parental conflict, SPB, all three types of bullying behaviours and gender. The socioeconomic background variables were no longer significant.

SBP and parental conflict accounted for part of the relationship between “bullies”, “bully-victims”, and self-harm, as the odds ratio for self-harm decreased more than 10% from model 5 to model 6 for “bully-victims” and “bullies”. Parental conflict also accounted for part of the relationship between the “bullied” and self-harm, as the OR decreased from 2.2 (CI 1.9–2.6) to 2.0 (CI 1.7–2.4) when only parental conflict (and not SBP) was included in the model.

### Protective factors

Table 3, Model 1 displays the OR for self-harm for the various possible protective factors in bivariate analyses. Parental support and friendship were the strongest protective factors for self-harm. The odds ratio for gender, again, remained quite stable after controlling for different predictors (models 1–6). We controlled for mother- and father’s education and family’s financial situation in models 3–6. The mother’s education and family’s financial situation was still significantly related to self-harm after controlling for gender and bullying behaviour (Model 3). Conversely, father’s education was not significant.

**Table 3:** OR of self-harm last year on bullying behaviour, demographic-, parental-, friend- and school variables

	Model 1	Model 2	Model 3	Model 4	Model 5	Model 6
	OR (95% CI)	OR (95% CI)	OR (95% CI)	OR (95% CI)	OR (95% CI)	OR (95% CI)
Neither bullied nor bullies	1.0 (ref.)					
Bullied	5.04 (4.48–5.68)***	4.94 (4.36–5.59)***	4.27 (3.68–4.95)***	3.69 (3.16–4.31)***	3.62 (3.10–4.24)***	2.42 (2.05–2.86)***
Bullies	3.19 (2.48–4.10)***	4.43 (3.40–5.77)***	3.60 (2.60–4.98)***	2.40 (1.70–3.41)***	2.37 (1.6–3.37)***	1.89 (1.31–2.73)***
Bully-victims	5.97 (4.86–7.35)***	8.29 (6.65–10.34)***	7.90 (6.02–10.37)***	5.60 (3.90–7.02)***	5.44 (4.05–7.29)***	3.48 (2.56–4.73)***
Gender	3.34 (3.02–3.71)***	3.73 (3.34–4.17)***	3.51 (3.08–4.01)***	3.94 (3.43–4.53)***	3.94 (3.42–4.54)***	3.71 (3.22–4.28)***
Family’s financial sit.	0.62 (0.60–0.65)***		0.72 (0.68–0.76)***	0.82 (0.77–0.87)***	0.82 (0.76–0.87)***	0.89 (0.83–.95)***
Education mother	0.69 (0.61–0.78)***		0.78 (0.68–0.90)**	0.88 (0.75–1.02)	0.87 (0.75–1.02)	0.88 (0.76–1.03)
Education father	0.84 (0.74–0.94)***		0.99 (0.86–1.14)	1.04 (0.89–1.20)	1.04 (0.90–1.20)	1.05 (0.91–1.22)
Parental support	0.20 (0.18–0.20)***			0.30 (0.26–0.34)***	0.31 (0. 26–0.35)***	0.37 (0.32–0.42)***
Parental monitoring	0.81 (0.79–0.82)***			0.88 (0.86–0.90)***	0.88 (0.86–0.91)***	0.91 (0.89–0.94)***
Friend support	0.63 (0.55–0.71)***				1.09 (0.90–1.33)	1.22 (1.00–1.50)*
Friendship	0.46 (0.41–0.53)***				0.74 (0.61–0.90)**	0.89 (0.73–1.08)
School well-being	0.73 (0.72–0.74)***					0.82 (0.80–0.84)***

Note: *p < 0.05 **p < 0.01 ***p < 0.001. *OR* Odds ratio, Family’s financial sit. = Family’s financial situation. Model 1 shows crude OR, bivariate associations with no controls, Model 2 shows adjusted OR with control for bullying behaviour and gender, Model 3 shows adjusted OR with control for bullying behaviour, gender, high family financial situation and high mother and father education. Model 4 shows adjusted OR with control for bullying behaviour, gender, high family financial situation, high mother and father education, parental support, and parental monitoring, Model 5 shows adjusted OR with control for bullying behaviour, gender, high family financial situation, high mother and father education, parental support, parental monitoring, friendship and friend support, Model 6 shows adjusted OR with control for all independent variables in the table (bullying behaviour, gender, high family financial situation, high mother and father education, parental support, parental monitoring, friendship, friend support, and school well-being)

In model 4, we found that both parental support and parental monitoring had a strong protective effect on self-harm after controlling for bullying behaviour, gender and socioeconomic background. The odds ratio for the groups of bullying behaviour was reduced more than 10% from Model 3 to Model 4, indicating that parental support and parental monitoring accounted for part of the relationship between self-harm and bullying behaviour. Mother’s education was not significantly related to self-harm in Model 4 to 6, but good family financial situation was significant in Model 3 to 6.

When friend support and friendship were included in Model 5, the changes in OR for all bullying groups from Model 4 to 5 were minimal. However, when we added school well-being in Model 6, the OR for the “bullied” and “bully-victims” decreased by more than 10%. This indicates that school well-being accounted for part of the relationship between self-harm and the groups “bullied” and “bully-victims”.

Parental support was the strongest protective factor for self-harm after controlling for all factors (Model 6). Additionally, parental monitoring, school well-being and a good family financial situation was significant in the final model.

### Interaction effects

**Fig. 1:**
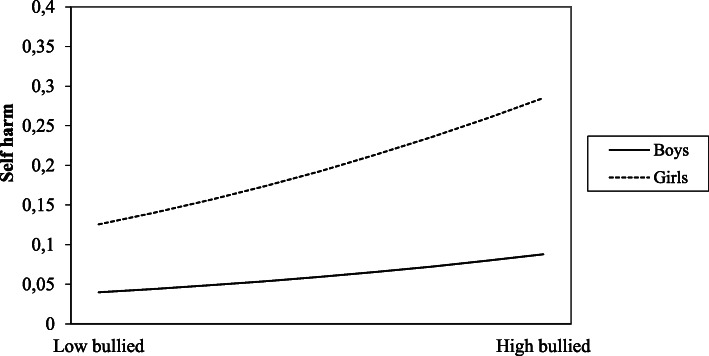
Interaction effect between gender (boys and girls) and the “bullied” on self-harm. Note: Low bullied = not bullied, High bullied = bullied

The odds of self-harm among girls was 3.3 times greater (OR = 3.34) than among boys (see Table 4, Model 1). In Model 3, we tested for interaction effects between gender and bullying behaviour. There was a positive significant interaction effect (*p* = 0.04) between gender and the “bullied” group (see Table 4, Model 3). This indicates that the risk of self-harm for the “bullied” compared with those who were “neither bullied nor bullies” was significantly greater for girls than for boys, as shown in Table 4 and Fig. 1.

**Table 4:** Interaction effects of self-harm on bullying behaviour and gender, school well-being, parental-, and friend support

	Model 1 OR (95% CI) Bivariate effect	Model 2 OR (95% CI) Multivariate effect	Model 3 OR (*b*) Interaction effects
Neither bullied nor bullies	1.0 (ref.)		Gender*
Bullied	5.04 (4.48–5.68)***	4.94 (4.36–5.59)***	1.32 (0.28)*
Bullies	3.19 (2.48–4.10)***	4.43 (3.40–5.77)***	0.84 (−0.18)
Bully-victims	5.97 (4. 86–7.35)***	8.29 (6.65–10.34)***	0.68 (− 0.39)
Gender	3.34 (3.02–3.71)***	3.73 (3.34–4.17)***	
Neither bullied nor bullies	1.0 (ref.)		Parental support*
Bullied	5.04 (4.48–5.68)***	4.38 (3.85–4.97)***	1.35 (0.30)*
Bullies	3.19 (2.48–4.10)***	2.40 (1.83–3.15)***	1.24 (0.22)
Bully-victims	5.97 (4.86–7.35)***	4.83 (3.85–6.07)***	1.08 (0.08)
Parental support	0.20 (0.18–0.22)***	0.23 (0.21–0.26)***	
Neither bullied nor bullies	1.0 (ref.)		School well-being*
Bullied	5.04 (4.48–5.68)***	2.60 (2.28–2.97)***	1.06 (0.06)**
Bullies	3.19 (2.48–4.10)***	1.98 (1.50–2.62)***	1.01 (0.01)
Bully-victims	5.97 (4.86–7.35)***	2.63 (2.08–3.32)***	1.09 (0.09)**
School well-being	0.73 (0.72–0.74)***	0.76 (0.75–0.78)***	
Neither bullied nor bullies	1.0 (ref.)		Friend support*
Bullied	5.04 (4.47–5.68)***	4.93 (4.36–5.56)***	1.68 (0.52)**
Bullies	3.19 (2.48–4.09)***	3.10 (2.40–4.01)***	0.78 (−0.25)
Bully-victims	5.97 (4.86–7.35)***	6.13 (4.96–7.59)***	0.70 (−0.35)
Friend support	0.63 (0.55–0.71)***	0.80 (0.70–0.92)**	

Note. *p < 0.05 **p < 0.01 ***p < 0.001. Self-harm = Self harmed last 12 months. Model 1 shows the crude OR, the bivariate associations with no controls, Model 2 shows adjusted OR with control for gender or the potential protective variables: parental support, school well-being and friend support. Model 3 shows the regression analysis with the interaction terms: bullying behaviour multiplied by gender, and bullying behaviour multiplied by the potential protective variables (parental support, school well-being and friend support). Only significant results reported

Furthermore, we tested for interaction effects between the three bullying behaviours and all protective factors on self-harm (see Table 4). Only significant interaction effect is shown in Table 4. The interactions between parental support and the “bullied”, and between school well-being and the “bullied” and the “bully-victims” were significant (see Table 4, Model 3).

**Fig. 2:**
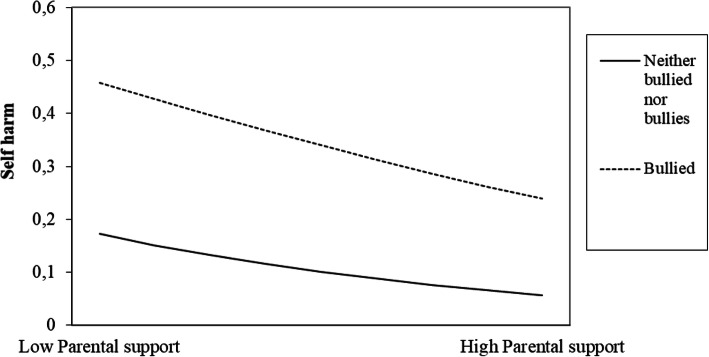
The figure shows the interaction effect between “Bullied” times “Parental support” on self-harm

**Fig. 3:**
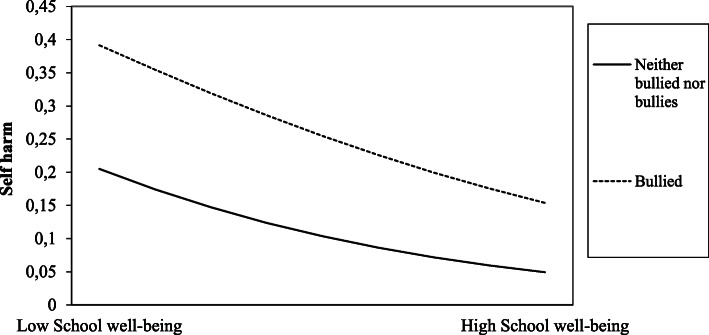
Interaction effect between school-well-being and the “bullied” on self-harm

**Fig. 4:**
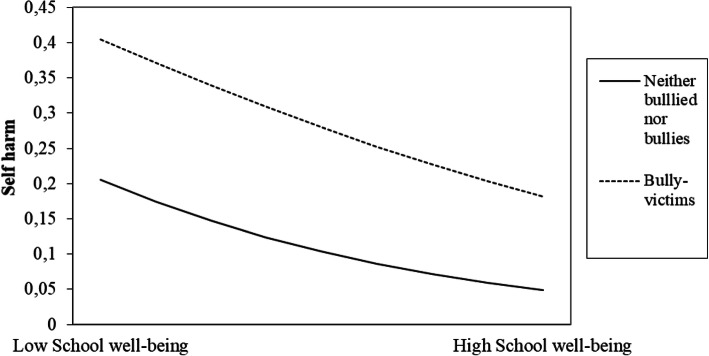
Interaction effect between school well-being and the “bully-victims” on self-harm

We then plotted the unstandardized regression beta-coefficients of the bulling behaviours, the protective factors and the interactions, in order to aid interpretation (see Fig. 1, 2, 3, 4, 5 for the results). In Fig. 2, the regression line for parental support is steeper for the “bullied” than for the “neither bullied nor bullies” group. Thus, parental support was more protective of self-harm among the “bullied” compared to the “neither bullied nor bullies” group. Similarly, the regression line for school-well-being is steeper for the “bullied” (Fig. 3) and for the “bully-victims” (Fig. 4) than for “neither bullied nor bullies”. Thus, school well-being was more protective of self-harm among the “bullied” and the “bully-victims” than for those who were not bullied nor bullies.

**Fig. 5:**
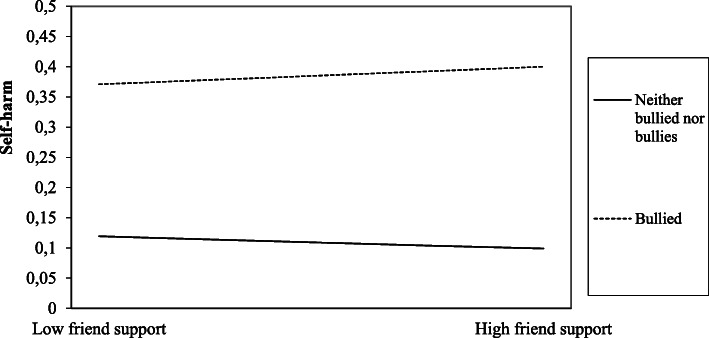
Interaction effect between friend support and the “bullied” on self-harm

Lastly, we found a significant interaction effect between “friend support” and the “bullied” (see Table 4 and Fig. 5). The result (see Fig. 5) shows that friend support was slightly less protective of self-harm among the “bullied” compared to the “neither bullied nor bullies”.

## Discussion

The aim of the study was to examine the relationship between self-harm and bullying, both being bullied and bullying others, and factors that may contribute to this relationship. The odds of self-harm were six times higher for the “bully-victims”, five times higher among the “bullied” and three times higher among the “bullies”, compared to adolescents who were neither bullied nor bullies. The increased risk of self-harm among these groups is previously documented in studies [8, 9, 20, 30, 31]. Our study also confirms that bully-victims are especially vulnerable [30]. They are considered to have the broadest range of adjustment problems, presenting difficulties common to both bullies and victims [30, 51], and are high in both externalizing- and internalizing problems. Barker [30] found that many of the bully-victims first have a history of being bullied, and then begin to bully peers later in adolescence. They suffer from both emotional problems and aggression.

### Risk factors

As hypothesised, the odds of self-harm were higher among adolescent girls compared to boys. This is consistent with earlier studies [3, 6, 7]. The gender difference remained after controlling for the three types of bullying behaviour. Additionally, we found a significant interaction effect between gender and “being bullied”, meaning that the odds of self-harm in the face of being bullied was significantly greater for girls than boys, compared with their “neither being bullied nor bullying” counterparts. We are not aware of any previous research which shows this, and thus the reasons for this gender difference should be investigated in future research. However, one explanation may be that girls have a greater susceptibility to stress in social relationships compared to boys [52, 53]. Another reason could be that girls are twice as likely as boys to experience depression during adolescence [54] and depression is associated with both being bullied and self-harm [25].

Symptoms of depression and anxiety accounted for the largest part of the relationship between self-harm and being bullied, and some of the relationship between self-harm and the “bully-victims”, and the bullies. Thus, the association between being bullied and self-harm appears to be confounded by depression and anxiety. This is consistent with earlier studies [9, 19, 20, 27–29]. Although our data are cross-sectional and we cannot be sure about causality, earlier longitudinal studies suggest that depressive symptoms mediate the relationship between being bullied and self-harm [29].

Parental conflict was associated with an increase in the odds of self-harm for all three bullying groups. This may be because negative relationships with parents, or ongoing inter-parental conflicts, might lead to deficits in emotion regulation and thus increase the likelihood of self-harm [55, 56]. The present study is one of a few considering parental conflict as an explanation of the relationship between bullying behaviour and self-harm. An exception was a large study of McMahon [10] among adolescent boys. This study showed that conflicts with parents and school-related problems heightens the risk of self-harm among boys who were bullied. This study did not, however include girls, and did not include the link between bullies and self-harm, and bully-victims and self-harm, as the present study did.

Interestingly, low socioeconomic background had a significant association with self-harm when controlling for bullying behaviour, but that it ceased to be significant after controlling for parental conflict. Thus, conflicts both with, and between, parents may be more important for self-harm than one’s socioeconomic background.

School behavioural problems was an important risk factor for self-harm and accounted for some of the relationship between self-harm and the bullies, and self-harm and bully-victims in the present study. It is possible that the act of bullying is part of a broader concept of externalizing behaviour that includes conduct behavioural problems with aggressive and delinquent behaviours, school failure, and drop out [51].

### Protective factors

Parental monitoring was a significant protective variable of self-harm, also after controlling for bulling behaviours and all covariates. However, there was no significant interaction effect between parental monitoring and bullying behaviour. Thus, our study suggests that parental monitoring does not buffer against self-harm when being bullied. This is in contrast to an earlier study by Hay and Meldrum [9]. This mismatch in findings could be due to the way authoritative parenting was measured. Hay & Meldrum [9] included three dimensions of authoritative parenting: supervision/parental monitoring, fair discipline, and parent–child involvement, while our measure mostly included the supervision/monitoring part of the measure. Another explanation may be due to cultural differences in parenting style. Garcia and Garcia for instance, found that the most optimal parenting style for adolescent mental health may be cultural specific [57].

Parental support had a significant protective effect on self-harm among boys and girls, even after controlling for the three types of bullying behaviour. Furthermore, we found a significant interaction effect between parental support and the “bullied” group. Thus, parental support appears more protective of self-harm for those who are bullied compared to those who are not. This is in line with some earlier studies [9, 20].

Friendship and friend support had a significant protective bivariate association with self-harm in the present study. However, friend support was no longer significant associated with self-harm after controlling for bullying behaviour and parental support. In addition, friendship and friend support accounted for very little of the relationship between self-harm and the three types of bullying behaviour. Friend support was even less protective of self-harm among those who were bullied, compared to the “neither bullied nor bullies”. A review study examining the impact of social modelling on self-harm, showed that exposure to self-harm through peers may for some adolescents, contribute to onset and maintenance of self-harm [58]. A possible explanation for the finding that friend support was less protective of self-harm among those who were bullied, compared to those who were not bullied or bullying, could be that adolescents do not receive adequate support from friends when dealing with difficult emotional issues related to self-harm and bullying. Those adolescents who are both being bullied and who self-harm may be extra vulnerable and will need support from adults such as parents or adults at school.

The present study found a significant interaction effect between school well-being and the “bullied”, and school well-being and the “bully-victims”. Thus, school well-being (including support from teacher) was more protective of self-harm for the bullied and the bully-victims, than it was for the “neither bullied nor bullies”. Previous research suggests that related measures of school well-being (school connectedness, teacher support, liking school) was protective of self-harm among adolescents [19]. However, the present study appears to be the first investigating the buffering effect of school well-being on bullying behaviour and self-harm among adolescents. Thus, this might be an important result for the prevention of self-harm. Schools, parents, and health care professionals should be aware of the importance of school well-being for adolescents who are being bullied, in terms of identifying those at risk of self-harm.

### Strengths and limitations

This study is based on a large population of adolescents (*N* = 16,182) with a high response rate (87%) and thus, representative of the in-school adolescent population. Furthermore, this study is one of few that looks at differences in factors that moderate the relationship between different types of bullying behaviour and self-harm. No previous studies have investigated the moderation effect of school well-being, on bullying behaviour and self-harm among adolescents. This is an important strength of the study and could pave the ground for future research and school-based self-harm intervention studies.

The present study has some limitations. Firstly, our data were cross-sectional, which does not allow causal interpretations to be drawn. Thus, the relationships between our predictor variables (such as bulling) and self-harm are associations and cannot demonstrate cause-effect. However, previous longitudinal studies found that bullying increases the risk of self-harm and not the other way around [29]. Secondly, the measures in our study are based on self-report, which may be biased because people tend to give socially desirable answers. However, earlier studies have shown that self-report measures are usually reliable [59].

Thirdly, our measures of self-harm and bullying are not standardised, since the survey was not specifically designed for assessing self-harm. Consensus of the definition of self-harm is however lacking in the literature and adolescents may have different understandings of what is meant by self-harm. Nonetheless, 15% reported having engaged in self-harm in the previous 12 months, which is consistent with earlier studies finding the 12-month prevalence rate to be between 10 to 19% around the world [3–5]. This lends support to the validity of the findings.

Fourthly, a limitation could be that we included cyberbullying and traditional bullying in the same measure. However, we conducted a sensitivity test, to test if the result of the logistic regression analysis were different if cyberbullying was excluded from the measure of bullying. Results showed very small differences, most likely because only cyberbullying (without traditional bulling) was not common (e.g., less than 7% of those who reported being bullied). This supports earlier studies that found that over 85% of those involved in cyberbullying were also involved in traditional bullying [60], and two studies of bullying among adolescents found that only 1% reported being only cyber victims [61, 62].

### Practical implications

Based on our findings, we see some practical implications. First, it is important to focus on both the bullied, the bullies and the bully-victims when developing interventions. Second, it also seems necessary to teach both those who are bullied and those who bully how to cope with depression and anxiety, such as learning adequate emotion-regulation skills, to prevent self-harming behaviour. Third, it may be helpful to include family communication in possible interventions that target bullying, as these factors seem to protect both the bullied, the bullies and bully-victims from engaging in self-harm. Fourth, school-based strategies such as improving school well-being and relationships at school may be an important element in the prevention of youth self-harm behaviours and bullying. Finally, interventions that foster nurturing environments, both at school and at home, may be effective for preventing the development of psychological problems and bullying behaviour [63]. Prevention efforts should also be aware of peer networks that could have a negative influence on adolescents who self-harm. This is because strategies simply focusing on promoting friend support may not benefit youth with respect to self-harm and bullying, unless friends have the ability and maturity necessary to function as a positive influence.

## Conclusion

Self-harm is not uncommon among adolescents and bullying is an important risk factor. Our study shows that it may have a greater effect on girls than on boys. Depression, anxiety and parental conflict accounted for some of the relationship between being bullied and self-harm, indicating the importance of family environment and emotion regulation. School-behavioural problems on the other hand is an important risk factor for self-harm for bullies and bully-victims. Our study highlights that parental support and school well-being may moderate the harmful consequences of bullying behaviour on self-harm among adolescents.

## Data Availability

The dataset used (Ungdata) during the current study are available upon reasonable request from the Norwegian Social Research Institute (NOVA), (http://www.ungdata.no/English).

## Abbreviations

HSCL: Hopkins symptom checklist
PCA: Principal components factors analysis
SBP: School behavioural problems
OR: Odds ratio
DSH: Deliberate self-harm
NSSI: Non-suicidal self-injury
NOVA: Norwegian social research institute
NSD: Norwegian centre for research data
